# Near-Infrared Spectroscopy (NIRS) to Assess Infection Complications During the Acute Phase of Acute Pancreatitis

**DOI:** 10.3390/diagnostics14232647

**Published:** 2024-11-24

**Authors:** Nobutaka Chiba, Tsukasa Yagi, Minori Mizuochi, Jun Sato, Takeshi Saito, Atsushi Sakurai, Kosaku Kinoshita

**Affiliations:** 1 Division of Emergency and Critical Care Medicine, Department of Acute Medicine, Nihon University School of Medicine, Tokyo 173-8610, Japan; chiba.nobutaka@nihon-u.ac.jp (N.C.); orita.minori@nihon-u.ac.jp (M.M.); sato.jun@nihon-u.ac.jp (J.S.); saito.takeshi@nihon-u.ac.jp (T.S.); sakurai.atsushi@nihon-u.ac.jp (A.S.); 2 Division of Cardiology, Department of Medicine, Nihon University School of Medicine, Tokyo 173-8610, Japan; yagi.tsukasa@nihon-u.ac.jp

**Keywords:** microcirculation, oxygen consumption, peripheral vasodilatory capacity, arterial lactate, monitoring

## Abstract

Background: Acute pancreatitis (AP) severity is correlated with systemic infection incidence in the acute phase, and it is important to assess inflammation during the disease course and to recognize infection at an early stage. As in sepsis, inflammation in AP impairs tissue oxygen metabolism and disrupts microcirculation. We performed a vascular occlusion test (VOT) via near-infrared spectroscopy (NIRS), which noninvasively monitors local oxygen in peripheral tissues, to evaluate tissue oxygen metabolism and blood circulation during the acute AP phase. Methods: Tissue oxygen metabolism was measured via an NIRS probe attached to the thenar eminence at admission and 7 days after admission. The upper arm was wrapped with a sphygmomanometer cuff while avoiding brachial artery compression for 3 min. The minimum desaturation value was defined as the minimum tissue oxygen index (TOI), the maximum reactive hyperemia value after release was defined as the maximum TOI, and the difference was defined as the ∆TOI. The time from the minimum TOI to maximum TOI was defined as the TOI interval. Results: Fifteen healthy volunteers, 13 patients with AP, and 12 patients with sepsis were included. The TOI at baseline and ∆TOI (parameter describing tissue oxygen metabolism) decreased in a stepwise manner, and the TOI interval (measure of peripheral vasodilatory capacity) was protracted in a stepwise manner among the three groups. In a subgroup analysis, no significant differences in the NIRS-derived variables between patients with AP complicated by infection and those without infection were observed at admission; however, after 7 days, the groups significantly differed. Additionally, blood lactate concentrations were significantly correlated with the ∆TOI and TOI. Conclusions: Mild tissue oxygen metabolism impairment and tissue perfusion occurred in AP compared with sepsis, and changes similar to those in sepsis occur in AP complicated by infection. Further research is needed to evaluate whether these values can be applied to treating this group of patients.

## 1. Introduction

Acute pancreatitis (AP) is not simply a local inflammatory disease that is limited to pancreatic and peripancreatic tissues, it can present in its most serious form as systemic inflammatory response syndrome (SIRS), multiple organ failure, sepsis, and death [1]. The disease is self-limiting and mild in the majority of patients; however, in up to 20% of patients, the disease is severe due to local and systemic complications [2]. The mortality of AP has two significant events, with organ damage occurring in the early stages and infection occurring in the late stages [3]. Therefore, it is important to assess inflammation during AP and recognize the onset of infection at an early stage. As occurs in sepsis, inflammation in AP causes endothelial dysfunction, tissue hypoperfusion, microcirculatory disturbances, and peripheral circulatory disturbances [4,5,6]. By monitoring these conditions, it may be possible to identify the onset of complications (such as infection) and link this onset to treatment.

Although information on tissue oxygen metabolism and oxygen delivery has long been obtained via the insertion of a central venous catheter or pulmonary artery catheter [7], a simple bedside measurement method is needed. One such method is NIRS, which enables local oxygen monitoring in peripheral tissues [8]. The principle of NIRS involves the use of a technology that transmits near-infrared light of 680–800 nm from the NIRS probe and noninvasively measures the light attenuation signal caused by hemoglobin in microvessels in the tissue. NIRS monitors only vessels with a diameter < 1 mm (such as arterioles, capillaries, and venules) [9]. The brain [10], kidneys [11], and skeletal muscles [12] represent sites where NIRS monitoring can be performed at the bedside. Based on different absorption spectra, NIRS measures oxyhemoglobin (oxy-Hb) and deoxyhemoglobin (deoxy-Hb) passing through blood vessels and can measure tissue oxygen saturation (StO_2_), which is the ratio of oxygenated hemoglobin to total hemoglobin (total-Hb, which is the summation of oxy-Hb and deoxy-Hb) in the tissue. StO_2_ is also referred to as the tissue oxygenation index (TOI) or regional oxygen saturation (rSO_2_), depending on the measurement device that is used. NIRS monitoring in clinical settings is used to measure cerebral oxygenation and renal blood flow during the perioperative period, particularly during cardiac and carotid artery surgeries [8]. In addition, by measuring the temporal changes in oxy-Hb and deoxy-Hb, it is also possible to estimate oxygen consumption based on changes in the oxygen concentration in tissues and to evaluate tissue oxygen metabolism, tissue perfusion, and vasodilation capacity in real time [9]. These methods have been used to evaluate tissue oxygen metabolism and tissue perfusion during sepsis [13]. In pancreatic diseases, NIRS is used in the evaluation of blood flow at anastomotic sites during surgery and in the evaluation of postoperative complications [14,15]. These results make it possible to evaluate tissue oxygen metabolism and blood flow in the pancreas. However, no study has reported the evaluation of tissue oxygen metabolism and tissue perfusion in peripheral tissues in AP, in which local inflammation progresses to systemic inflammation. Therefore, we aimed to investigate the relationship between inflammation and infection by measuring tissue oxygen metabolism and tissue perfusion via NIRS (NIRO-200NX, Hamamatsu Photonics, Hamamatsu, Japan)-derived variables during AP in the acute phase.

## 2. Methods

### 2.1. Study Design and Patients

The Clinical Research Review Committee of Nihon University Hospital approved the study protocol, and informed consent (approval number 161202) was obtained following the ethical principles of the Declaration of Helsinki. This study was designed as a single-institution prospective observational investigation; additionally, consecutive patients with acute pancreatitis and sepsis who were admitted to the intensive care unit (ICU) and healthy volunteers who consented to participate in the study among the hospital’s medical staff were enrolled from April 2019 to March 2022. In this study, demographic data, clinical characteristics, and medical data from patients’ electronic medical records were reviewed.

The diagnostic criteria for AP were based on two or more of the following conditions: (1) acute onset of upper abdominal pain, (2) serum amylase or lipase activity three times greater than normal, and (3) findings on cross-sectional abdominal imaging consistent with AP [3]. To ensure that the patients were eligible for the study, those with pancreatic tumors or immune diseases who were admitted to the hospital more than 48 h after the onset of symptoms and those with incomplete laboratory examination results were excluded. The severity of disease was determined by using the criteria of the Japanese Ministry of Health, Labor and Welfare Study Group for Acute Pancreatitis (2008) (Japanese Severity Score [JSS]) when the total prognostic factor score was 3 points or greater or when the computed tomography grade was 2 or greater [16]. Computed tomography (CT) scans were evaluated by a radiologist. Sepsis was defined as infection plus systemic manifestations of infection [17].

Postdiagnosis treatments included intravenous fluid administration within 24 h to achieve a mean arterial pressure (MAP) of 65 mmHg or higher and a urine output of 0.5 mL/kg/h. SAP patients were administered enteral nutrition (EN) within 48 h, dialysis in cases of renal failure, mechanical ventilation, prophylactic antibiotics, and protease inhibitors. Analgesics were administered when necessary. Patients with sepsis received standard treatment for localized infection, severe sepsis, and septic shock, including source control, fluid infusion, catecholamine infusion, organ failure replacement and/or support therapy, and intensive control of blood glucose.

### 2.2. Measurement Methods

**Figure 1:**
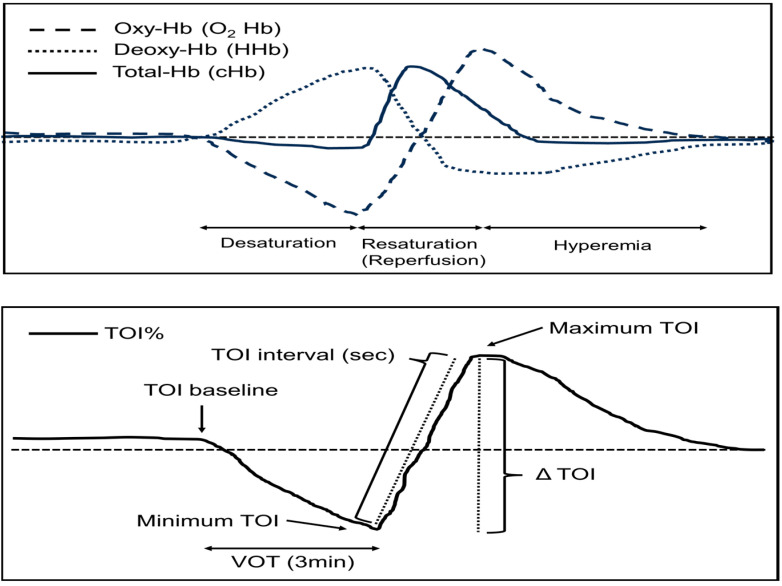
A representative near-infrared spectroscopy dynamic variable curve. Total-Hb: total hemoglobin, oxy-Hb: oxyhaemoglobin, deoxy-Hb: deoxyhaemoglobin, TOI: tissue oxygen index, VOT: vascular occlusion test.

The VOT is a method of blocking blood flow to the muscles via a tourniquet for a fixed period of time (for example, 3 min) to cause tissue desaturation. During VOT, the Oxy-Hb level decreases due to occlusion of the arteries, and venous congestion occurs due to occlusion of the veins, thus causing an increase in deoxy-Hb (Figure 1). When the tourniquet is released and blood flow is restored, a reactive hyperemic response occurs, which represents the ability of the tissue to self-regulate blood flow and oxygenation. Therefore, the rate of recovery of StO_2_ represents the strength of the reactive hyperemic response. The rate at which the tissue is reoxygenated represents the reserve capacity and functionality of the endothelium, mitochondria, and microcirculation. If self-regulating ability is not impaired, blood flow will quickly recover, thus resulting in a steep recovery gradient. Conversely, if there is functional disorder, reoxygenation will be impaired, and the recovery gradient will be shallower. Therefore, the use of NIRS in combination with the VOT is considered a method for evaluating endothelial cell function, microcirculatory capacity, and self-regulating reserve capacity [18,19].

### 2.3. Study Protocol

In the present study, we used the NIRS-measured TOI to evaluate tissue oxygenation, which is compatible with tissue oxygen saturation (StO_2_). The NIRS probe was attached to the right thenar eminence; moreover, after the upper arm was wrapped with a sphygmomanometer cuff while avoiding compression of the brachial artery, the initial oxy-Hb and deoxy-Hb values were measured, and the calculated TOI was defined as the TOI baseline. The brachial artery blood flow was then blocked for 3 min by inflating the cuff to 50 mm Hg above the systolic arterial pressure (which represented the VOT). The minimum value of desaturation was defined as the minimum TOI (tissue oxygen index), and the maximum value of reactive hyperemia after release was defined as the maximum TOI. The difference between the two parameters was defined as the ∆TOI. The time from the minimum TOI to the maximum TOI was defined as the TOI interval (Figure 1). NIRS measurements via the VOT and all of the blood sampling were performed within 2 h of admission and 7 days after admission. Blood samples were collected from the arterial line in the left radial artery before the VOT. The utilized parameters included complete blood count; C-reactive protein (CRP) and lactate dehydrogenase (LD), which are markers of systemic inflammation; and lactate, which is a marker of tissue oxygen metabolism. Data including patient age and sex were collected at the time of admission; additionally, the following prognostic scores were collected at the time of admission [20]: (1) Acute Physiology and Chronic Health Evaluation II (APACHE II) score and (2) Sequential Organ Failure Assessment (SOFA) score. Hemodynamic measurements, including heart rate, systolic blood pressure, diastolic blood pressure, and mean arterial pressure (MAP), were also obtained from healthy volunteers. We did not obtain blood samples from these patients.

We compared the changes in the TOI according to the VOT and NIRS-derived variables in healthy volunteers and patients with acute pancreatitis and sepsis at admission. In the second analysis, due to the fact that one of the complications of AP is infection [2], we evaluated the changes in the TOI at admission and on day 7 after admission in AP patients with infection complications compared with those without infection. We defined infection as the presence of bacteria based on blood cultures or local cultures obtained and evaluated via CT scan. Pancreatic-related infections were confirmed via percutaneous, image-guided, or endoscopic fine-needle aspiration or the presence of extraluminal gas in the pancreatic and/or peripancreatic tissue on contrast-enhanced CT [16]. Finally, we evaluated the relationship between changes in the TOI by VOT and lactate levels in patients with AP at 7 days after admission.

### 2.4. Statistical Analysis

Statistical Production and Services Solution 22.0 (SPSS 22.0, SPSS Inc., Chicago, IL, USA) were used for statistical analysis. The data are presented as the means ± standard deviations (SDs), medians (interquartile ranges), or numbers of patients (%). A *p* value < 0.05 was considered to indicate statistical significance.

The differences between the two groups (AP and sepsis or AP with and without infection) were analyzed via Student’s *t* test or the Mann–Whitney U test for continuous variables and the χ^2^ test for categorical variables. Physiological data and NIRS parameters from the three groups were compared via one-way analysis of variance (ANOVA). The Tukey–Kramer test was subsequently performed. The correlation between lactate and NIRS parameters was examined via Spearman’s rank correlation coefficient.

## 3. Results

Fifteen healthy controls, 13 patients with AP, and 12 patients with sepsis were included in the study. The sources of infection in septic patients were bloodstream infections in four patients, soft tissue and myofascial infections in three patients, urinary tract infections in three patients, and pneumonia in two patients.

The cumulative data on the basic characteristics, as well as ICU and hospital lengths of stay, of patients and healthy volunteers are shown in Table 1. Age, heart rate, and MAP at admission were significantly different among the three groups. Compared with those of AP patients and sepsis patients, the APACHE II score, SOFA score, and lactate level were greater in septic patients, and the hemoglobin (Hb) level and hematocrit level were lower in this group. The ICU stay was shorter in septic patients compared to other patients.

**Figure 2:**
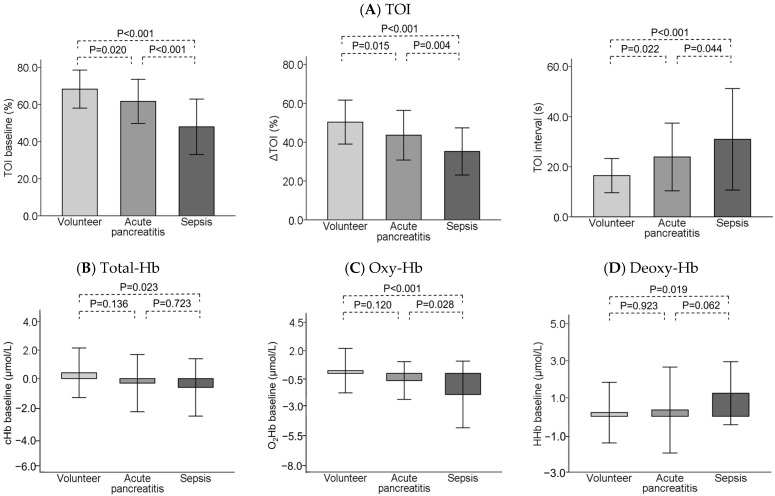
Comparison of NIRS-derived variables in healthy volunteers and patients with acute pancreatitis and sepsis at admission. TOI: tissue oxygen index, total-Hb: total hemoglobin, oxy-Hb: oxyhaemoglobin, deoxy-Hb: deoxyhaemoglobin.

NIRS-derived variables exhibited significant differences between each group with respect to the TOI baseline, ∆TOI, and TOI interval. The TOI baseline and ∆TOI decreased in a stepwise manner in the healthy volunteer, AP, and sepsis groups. The TOI interval was protracted in a stepwise manner in the three groups. NIRS-derived parameters (total Hb, oxy-Hb, and deoxy-Hb) were significantly different between patients with sepsis and healthy volunteers (Figure 2).

In a subgroup analysis, a comparison of patients with AP complicated by infection and those without complications by infection at admission and on the seventh day in the hospital is shown in Table 2. The sources of infection in patients with AP included generalized peritonitis in three patients, pneumonia in two patients, bloodstream infections in two patients, and ANC infection in one patient. At admission, the heart rate significantly differed between the two groups; however, on the seventh day, the heart rate, MAP, APACHE II score, SOFA score, SIRS score, Hb level, hematocrit level, LD level, CRP level, and lactate level significantly differed between the two groups.

**Figure 3:**
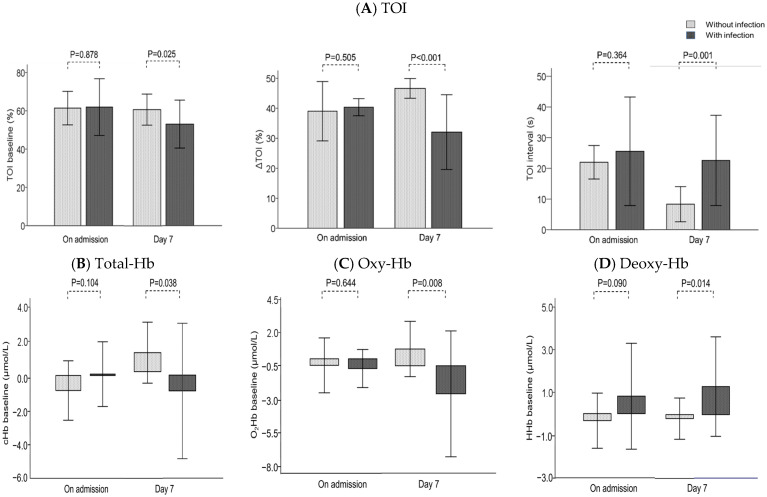
Comparison of NIRS-derived variables at admission and 7 days after admission in patients with acute pancreatitis complicated or uncomplicated by infection. TOI: tissue oxygen index, total-Hb: total hemoglobin, oxy-Hb: oxyhaemoglobin, deoxy-Hb: deoxyhaemoglobin.

**Figure 4:**
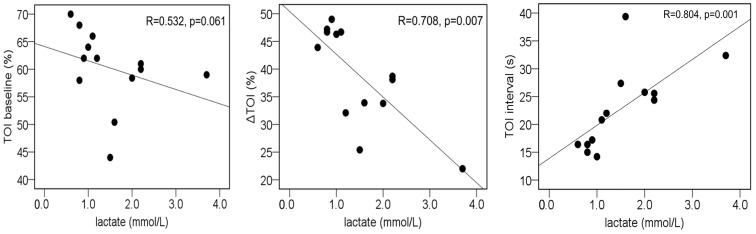
Relationship between lactate levels and near-infrared spectroscopy dynamic variables in patients with acute pancreatitis at 7 days after admission. TOI, tissue oxygen index.

The NIRS-derived variables at admission were not significantly different between the two groups; however, the variables at 7 days after admission were significantly different (Figure 3). In addition, regression analysis revealed that the blood lactate concentration was significantly correlated with the ∆TOI and TOI interval (Figure 4).

## 4. Discussion

One of the complications that can occur after the onset of AP is systemic infection, which is a cause of mortality [2]. We assessed tissue oxygen metabolism and tissue perfusion via NIRS, as we thought that cases of AP complicated by infection would have impaired tissue oxygen metabolism in the periphery compared with cases without infection. In the present study, NIRS measurements of the VOT in healthy volunteers, AP patients, and sepsis patients revealed stepwise changes in the TOI baseline, ∆TOI, and TOI interval values. Therefore, our results suggest that NIRS measurements may be used to assess tissue oxygen metabolism and blood circulation in both AP and sepsis. Furthermore, the evaluation of infectious complications of AP via measurements on the seventh day after the onset of illness revealed significant changes in the group with infectious complications. In AP, the determination of the development of infection is difficult because the inflammatory response is enhanced and inflammatory factors are elevated [21]. Therefore, NIRS measurements may be able to determine the onset of infection, which is one of the complications of AP. However, it has been reported that the systemic complications of AP are not limited to infections but can also include pulmonary, renal, and cardiocirculatory insufficiencies and coagulopathy [2]. In this study, the evaluation of tissue oxygen metabolism disorders in conditions with multiple complications was not performed. In addition, factors related to changes in total hemoglobin, such as age, cardiac output, hemoglobin levels, oxygen content, skeletal muscle mitochondrial content, and blood flow to skeletal muscle, affect oxygen delivery and oxygen consumption. In addition, when body temperature increases or pH decreases, the oxygen-Hb dissociation curve shifts in the direction of dissociation; thus, the amount of oxygen in the blood vessels decreases [22]. All of these values should be interpreted with caution, and further studies on this topic are warranted.

The causes of the decrease in TOI can be divided into three pathologies according to changes in hemoglobin [23,24]: (1) a decrease in oxy-Hb and an increase in deoxy-Hb reflect poor oxygenation or increased oxygen consumption; (2) an increase in total-Hb and an increase in deoxy-Hb reflect venous stasis; and (3) a decrease in total-Hb reflects ischemia or venoconstriction. In this study, the TOI baseline decreased in a stepwise manner in the healthy volunteer, AP, and sepsis groups. In contrast, no significant differences in NIRS-derived parameters were found between AP patients and healthy volunteers. Infusion loading is recommended for the early treatment of AP [2]. Targeted infusion resuscitation reportedly plays a role in increasing the systemic oxygen supply to support systemic oxygen consumption [25]. Therefore, it was suggested that the infusion load in the initial treatment may have contributed to the reduction in pathophysiology. However, a decrease in oxy-Hb and total-Hb was observed in AP complicated by infection on day 7. It has been reported that in AP, persistent inflammation causes fluid to flow into the third space, thus leading to a hypoosmolar state and microcirculatory disturbances [26]. Therefore, the persistence of inflammation due to infectious complications may result in decreased blood flow to the periphery and increased oxygen consumption. To support the increase in oxygen consumption, infusion or blood transfusion should be considered. In addition, the revised Atlanta classification recommends adequate sedation and analgesia [3]. Muscle oxygen consumption measured via NIRS is related to muscle activity [27] and decreases after sedation; therefore, it may be worth considering the use of these drugs.

In the present study, the VOT was also used for dynamic assessment to reflect tissue oxygen consumption and blood circulation due to reperfusion and hyperemia after ischemia by simultaneously occluding both arteries and veins [18]. Furthermore, this method has been reported for use in septic shock [25] and anesthesia [28], wherein static variables are unaffected despite circulatory disturbances. In severe cases of AP, organ damage and circulatory disturbances are known to occur [2]. Therefore, it is necessary to measure the VOT to evaluate tissue oxygen metabolism and tissue perfusion in AP, regardless of the degree of severity. The increase in total hemoglobin after VOTs represents the volume of blood that enters the monitored tissue bed, as the arterioles, capillaries, and venules maximally dilate to capacity during venous occlusion, and the StO2 recovery slope indicates the dilatation capacity of the peripheral blood vessels. It has been previously suggested that NO, which has vasodilatory properties, is involved in the StO2 recovery slope [25]. Similarly, excessive NO production due to inflammation has been reported in AP [29]. Therefore, the values indicated by the VOT could be used as indicators for continued infusion and the use of vasopressors during AP.

Elevated lactate has always been considered an indicator of hypoxia, which can reflect microcirculation and has been considered the “gold standard” [30]. Elevated lactate levels have been shown to reflect cellular dysfunction in sepsis [17], and an association between elevated lactate levels and impaired tissue oxygen metabolism has been reported [31]. The relationships between the ∆TOI and lactate in septic patients, as measured via NIRS, have been demonstrated to be negative [24]. Impaired tissue oxygen metabolism has also been shown to play an important role in the pathogenesis of AP [32]. It has been suggested that in the early stage of AP, systemic inflammatory syndrome causes fluid to flow into the third space, thus leading to hypo-osmoticaemia and microcirculatory disturbances; moreover, inadequate tissue perfusion and hypoxia may lead to elevated lactate levels associated with AP [26], and elevated lactate levels and decreased clearance during the course of the disease have been reported as being factors associated with poor prognosis [33]. In this study, both AP and sepsis patients had elevated lactate levels, and lactate levels were significantly greater in the group of patients with AP complicated by infection than in the uncomplicated group on day 7 of hospitalization. Furthermore, a correlation was observed between the lactate level and the ∆TOI or TOI interval. These findings suggest that complications of infection in AP cause further impairment of tissue oxygen metabolism. Factors involved in inflammation have been reported to indicate complications of pancreatic infection [34]. However, in cases in which it is difficult to determine whether the decrease in tissue oxygen metabolism is due to inflammation alone, the combination of NIRS variables, which can be noninvasively, easily, and continuously measured at the bedside, has the potential to detect pancreatitis-related infections at an early stage.

There are several limitations of this study. The major limitation is that this was a prospective observational study with a limited number of patients who were assessed at a single institution. In the future, research on larger patient groups will be necessary. In addition, the healthy volunteers who were included in this study were limited to hospital staff. Therefore, there is a possibility that the health characteristics of this group differed from those of the general population, and selection bias may have occurred. Second, NIRS is sensitive to variables such as skin pigmentation, fat tissue thickness, muscle mass, ambient light, and tissue edema [8,12]; however, these factors were not adjusted for in this study. In addition, the NIRS device that was used in this study was the NIRO-200NX, which is different from the devices used in other studies. Although the reproducibility is quite similar, the absolute values can differ depending on the NIRS device that is used [35]. The third limitation is that the Revised Atlanta Classification, which is generally used as a standard for evaluating the severity of acute pancreatitis 48 h after onset, was not used. However, in this study, due to the fact that treatment for AP requires early diagnosis and appropriate treatment of severe cases, the severity of the disease was assessed via the “Japanese classification”, which can determine the severity of pancreatitis within 48 h of onset. Therefore, if the assessment is based on the presence or absence of local or systemic complications after the onset of pancreatitis, different results may be obtained. However, the proportion of mild cases in the revised Atlanta classification was low in this study. In addition, this study excluded patients who had been hospitalized for 48 h or more at other hospitals. As complications of AP are assessed after 48 h, not all of the patients who developed complications were included in the study. Therefore, we were unable to assess all cases of infection that developed in patients with AP. This hospital is a regional core hospital and does not intensively admit patients with AP. The rate of severe pancreatitis was high because many patients were referred from other hospitals. In addition, although this study included patients with AP within 48 h of onset, the course of the disease until hospitalization varied. Therefore, the effect of prior treatment may be present because the measurements via NIRS were based on the date of admission and not on the date of onset. Finally, the NIRS measurements that were performed in this study were conducted after hospitalization and on the 7th day after onset, and this study only investigated the variables derived from NIRS before and after the VOT. Therefore, we did not conduct a long-term follow-up study to evaluate the real-time timing of treatment or improvements in tissue oxygen metabolism. However, NIRS is a device that continuously measures changes in the concentrations of oxy-Hb and deoxy-Hb in blood vessels. Therefore, further research is needed in the future.

## 5. Conclusions

Although there is mild impairment of tissue oxygen metabolism and tissue perfusion in AP compared with sepsis, similar changes to those that occur in sepsis are observed in AP complicated by infection. In cases in which it is difficult to determine whether the decrease in tissue oxygen metabolism is due to inflammation alone, the combination of NIRS variables, which can be noninvasively, easily, and continuously measured at the bedside, has the potential to detect pancreatitis-related infections at an early stage. Further research is needed to evaluate whether these values can be applied to the treatment of this group of patients.

## Figures and Tables

**Table 1:** Comparison of volunteers and patients with acute pancreatitis and sepsis.

	Volunteer (*n* = 15)	Acute Pancreatitis (*n* = 13)	Sepsis (*n* = 12)	*p* Value
(A) Characteristics				
Age	50.9 (7.1)	52.7 (16.6)	68.0 (10.7)	<0.001
Sex (male), *n* (%)	9 (60.0)	9 (69.2)	8 (66.7)	0.869
BMI	23.6 (2.7)	23.3 (3.5)	23.9 (4.9)	0.942
HR	70.8 (9.1)	93.8 (26.5)	80.9 (25.5)	0.026
MAP	99.2(14.1)	106 (21.9)	81.6 (16.4)	0.004
(B) Scoring				
APACHE II score, mean (SD)	N/A	9.77 (4.49)	17.00 (6.78)	0.006
SOFA score, median (IQR)	N/A	3 (1–4)	4.5 (2.5–8)	0.046
SIRS score, median (IQR)	N/A	2 (1–2)	2 (2–3)	0.225
(C) Blood sample, mean (SD)				
Hb (g/dL), mean (SD)	N/A	14.26 (2.00)	11.28 (2.56)	0.004
Hematocrit (%), mean (SD)	N/A	41.51 (6.93)	34.17 (7.70)	0.020
LD (U/L), mean (SD)	N/A	423.9 (245.9)	397.0 (293.6)	0.807
CRP (mg/dL), mean (SD)	N/A	12.86 (12.85)	16.32 (9.48)	0.450
Lac (mmol/L), mean (SD)	N/A	1.95 (0.75)	2.81 (1.17)	0.039
(D) Outcome				
ICU stay, median (IQR)	N/A	12 (10–16)	7 (5–9)	0.002
Hospital stay, median (IQR)	N/A	15 (12–39)	10 (6–16)	0.087

BMI: body mass index, HR: heart rate, MAP: mean arterial pressure, APACHE II: Acute Physiology and Chronic Health Evaluation II, SOFA: Sequential Organ Failure Assessment, SIRS: systemic inflammatory response syndrome, Hb: hemoglobin, LD: lactate dehydrogenase, CRP: C-reactive protein, Lac: lactate.

**Table 2:** Comparison of patients with acute pancreatitis with or without infection.

	On Admission			7 Days After Admission		
	Non-Infection	Infection	*p* Value	Non-Infection	Infection	*p* Value
Cases: numbers	6	7				
(A) Baseline characteristics						
Age	50.3 (13.4)	54.7 (19.9)	0.657			
Sex (male), *n* (%)	5 (83.3)	4 (57.1)	0.308			
BMI	24.7 (3.6)	22.1 (3.3)	0.205			
HR	78.3 (19.3)	107.1 (25.6)	0.046	73.33 (10.05)	88.86 (17.02)	0.008
MAP	115.2 (22.2)	98.1 (19.6)	0.400	101.33 (6.89)	77.86 (11.15)	0.001
(B) Scoring						
APACHE II score, mean (SD)	8.33 (2.25)	11.00 (5.68)	0.306	3.83 (2.48)	11.57 (3.45)	0.001
SOFA score, median (IQR)	1 (1–4)	4 (2–6)	0.101	0 (1–1.25)	2 (2–4)	0.005
SIRS score, median (IQR)	1.5 (0–2)	2 (2–4)	0.117	0 (0–0.25)	2 (1–3)	0.003
Prognostic factor, median (IQR)	0.5 (0–2)	4 (1–5)	0.092			
Revised Atlanta classification, *n* (%)			0.060			
Mild	3 (50.0)	0 (0.0)				
Moderate	2 (33.3)	2 (28.6)				
Severe	1 (16.7)	5 (71.4)				
(C) Blood sample, mean (SD)						
Hb (g/dL), mean (SD)	15.18 (2.44)	13.47 (1.20)	0.129	12.95 (1.55)	8.87 (0.62)	<0.001
Hematocrit (%), mean (SD)	38.12 (4.62)	45.46 (7.41)	0.064	37.78 (3.44)	26.61 (2.03)	<0.001
LD (U/L), mean (SD)	333 (219.34)	501.86 (255.91)	0.232	257.33 (97.49)	517.43 (226.03)	0.024
CRP (mg/dL), mean (SD)	8.14 (14.86)	16.91 (10.23)	0.235	8.65 (11.13)	21.95 (8.18)	0.031
Lac (mmol/L), mean (SD)	1.71 (0.57)	2.08 (1.03)	0.450	0.86 (0.17)	2.05 (0.81)	0.005

BMI: body mass index, HR: heart rate, MAP: mean arterial pressure, APACHE II: Acute Physiology and Chronic Health Evaluation II, SOFA: Sequential Organ Failure Assessment, SIRS: systemic inflammatory response syndrome, Hb: hemoglobin, LD: lactate dehydrogenase, CRP: C-reactive protein, Lac: lactate.

## Data Availability

The datasets that were analyzed during the current study are not publicly available because of contracts with hospitals that provide data to the database; however, the data are available from the corresponding author upon reasonable request.

## References

[1] Li H., Wu D., Zhang H., Li P. (2023). New insights into regulatory cell death and acute pancreatitis. Heliyon.

[2] Boxhoom L., Voemans R.P., Bouwense S.A., Bruno M.J., Verdonk R.C., Boemeester M.A., van Santvoort H.C., Besselink M.G. (2020). Acute pancreatitis. Lancet.

[3] Banks P.A., Bollen T.L., Dervenis C., Gooszen H.G., Johnson C.D., Sarr M.G., Tsiotos G.G., Vege S.S., Acute Pancreatitis Classification Working Group (2013). Classification of acute pancreatitis 2012: Revision of the Atlanta classification and definitions by international consensus. Gut.

[4] O’Reilly D.A., Kingsnorth A.N. (2001). A brief history of pancreatitis. J. R. Soc. Med..

[5] Van Laethem J.L., Marchant A., Delvaux A., Goldman M., Robberecht P., Velu T., Deviére J. (1995). Interleukin 10 prevents necrosis in murine experimental acute pancreatitis. Gastroenterology.

[6] Sandoval D., Gukovskaya A., Reavey P., Gukovsky S., Sisk A., Braquet P., Pandol S.J., Poucell-Hatton S. (1996). The role of neutrophils and platelet-activating factor in mediating experimental pancreatitis. Gastroenterology.

[7] Rodriguez A., Lisboa T., Martin-Loeches I., Diaz E., Trefler S., Restrepo M.I., Rello J. (2011). Mortality and regional oxygen saturation index in septic shock patients: A pilot study. J. Trauma.

[8] Scheeren T.W.L., Schober P., Schwarte L.A. (2012). Monitoring tissue oxygenation by near infrared spectroscopy (NIRS): Background and current applications. J. Clin. Monit. Comput..

[9] Nitzan M., Nitzan I., Arieli Y. (2020). The Various oximetric techniques used for the evaluation of blood oxygenation. Sensors.

[10] Wolf M., Ferrari M., Quaresima V. (2007). Progress of near-infrared spectroscopy and topography for brain and muscle clinical applications. J. Biomed. Opt..

[11] Owens G.E., King K., Gurney J.G., Charpie J.R. (2011). Low renal oximetry correlates with acute kidney injury after infant cardiac surgery. Pediatr. Cardiol..

[12] Creteur J., Carollo T., Soldati G., Buchele G., Backer D.D., Vincent J.L. (2007). The prognostic value of muscle StO_2_ in septic patients. Intensive Care Med..

[13] Shapiro N.I., Arnold R., Sherwin R., O’Connor J., Najarro G., Singh S., Lundy D., Nelson T., Trzeciak S.W., Jones A.E. (2011). for the Emergency Medicine Shock Research Network (EMSockNet). The association of near-infrared spectroscopy-derived tissue oxygenation measurements with sepsis syndromes, organ dysfunction and mortality in emergency department patients with sepsis. Crit. Care.

[14] Wakabayashi T., Barberio M., Urade T., Pop R., Seyller E., Pizzicannella M., Mascagni P., Charles A.L., Abe Y., Geny B. (2021). Intraoperative perfusion assessment in enhanced reality using quantitative optical imaging: An experimental study in a pancreatic partial ischemia model. Diagnostics.

[15] Sucher R., Scheuermann U., Rademacher S., Lederer A., Sucher E., Hau H.M., Brandacher G., Schneeberger S., Gockel I., Seehofer D. (2022). Intraoperative reperfusion assessment of human pancreas allografts using hyperspectral imaging (HIS). HepatoBliary Surg. Nutr..

[16] Takeda K., Yokoe M., Takada T., Kataoka K., Yoshida M., Gabeta T., Hirota M., Mayumi T., Kadoya M., Yamanouchi E. (2010). Assessment of severity of acute pancreatitis according to new prognostic factors and CT grading. J. Hepatobiliary Pancreat. Sci..

[17] Singer M., Deutschman C.S., Seymour C.W., Shakar-Hari M., Annane D., Bauer M., Bellomo R., Bernard G.R., Chiche J.D., Coopersmith C.M. (2016). The Third International Consensus Definitions for Sepsis and Septic Shock (Sepsis-3). JAMA.

[18] Lipcsey M., Woinarski N.C.Z., Bellomo R. (2012). Near infrared spectroscopy (NIRS) of the thenar eminence in anesthesia and intensive care. Ann. Intensive Care.

[19] Cortés D.O., Puflea F., Backer D.D., Creteur J., Vincent J.L. (2015). Near infrared spectroscopy (NIRS) to assess the effect of local ischemic preconditioning in the muscle of healthy volunteers and critically ill patients. Microvasc. Res..

[20] Szatmary P., Grammatikopoulos T., Cai W., Huang W., Mukherjee R., Halloran C., Beyer G., Sutton R. (2022). Acute Pancreatitis: Diagnosis and Treatment. Drugs.

[21] Rau B., Schilling M.K., Beger H.G. (2004). Laboratory markers of severe acute pancreatitis. Dig. Dis..

[22] Wood M.D., Jacobson J.A., Maslove D.M., Muscedere J.G., Boyd J.G., The Cerebral Oxygenation and Neurological Outcomes Following Critical Illness (CONFOCAL) Research Group (2019). The physiological determinants of near-infrared spectroscopy-derived regional cerebral oxygenation in critically ill adults. Intensive Care Med. Exp..

[23] Lima A., Van Bommel J., Sikorska K., Van Genderen M., Klijn E., Lesaffre E., Lnce C., Bakker J. (2011). The relation of near-infrared spectroscopy with changes in peripheral circulation in critically ill patients. Crit. Care Med..

[24] Soga T., Sakatani K., Yagi T., Kawamorita T., Yoshino A. (2014). The relationship between hyperlactatemia and microcirculation in the thenar eminence as measured using near-infrared spectroscopy in patients with sepsis. Emerg. Med. J..

[25] Skarda D.E., Mulier K.E., Myers D.E., Taylor J.H., Beilman G.J. (2007). Dynamic near-infrared spectroscopy measurements in patients with severe sepsis. Shock.

[26] Mederos M.A., Reber H.A., Girgis M. (2021). Acute pancreatitis. JAMA.

[27] Soares R.N., Mclay K.M., George M.A., Murias J.M. (2017). Differences in oxidative metabolism modulation induced by ischemia/reperfusion between trained and untrained individuals assessed by NIRS. Physiol. Rep..

[28] Futier E., Christophe S., Robin E., Petit A., Pereira B., Desbordes J., Bazin J.E., Vallet B. (2011). Use of near-infrared spectroscopy during a vascular occlusion test to assess the microcirculatory response during fluid challenge. Crit. Care.

[29] Wang G., Iv J.C., Wu L.F., Li L., Dong D.L., Sun B. (2014). From nitric oxide to hyperbaric oxygen: Invisible and subtle but nonnegligible gaseous signaling molecules in acute pancreatitis. Pancreas.

[30] Bruno R.R., Wernly B., Binneboessel S., Baldia P., Duse D.A., Erkens R., Kelm M., Mamandipoor B., Osmani V., Jung C. (2020). Failure of lactate clearance predicts the outcome of critically ill septic patients. Diagnostics.

[31] Pino R.M., Singh J. (2021). Appropriate clinical use of lactate measurements. Anesthesiology.

[32] Cuthbertson C.M., Christophi C. (2006). Disturbances of the microcirculation in acute pancreatitis. Br. J. Surg..

[33] Zeng J., Wan J., He W., Zhu Y., Zeng H., Liu P., Gong M., Liu F., Shao Q., Xia L. (2022). Prognostic value of arterial lactate metabolic clearance rate in moderate and severe acute pancreatitis. Dis. Markers.

[34] Chen X., Ning J., Li Q., Kuang W., Jiang H., Qin S. (2022). Prediction of acute pancreatitis complications using routine blood parameters during early admission. Immun. Inflamm. Dis..

[35] Yoshitani K., Kawaguchi M., Tatsumi K., Kitaguchi K., Furuya H. (2002). A comparison of the INVOS 4100 and the NIRO 300 near-infrared spectrophotometers. Anesth. Analg..

